# Experiences of Work-Family Conflict and Mental Health Symptoms by Gender Among Physician Parents During the COVID-19 Pandemic

**DOI:** 10.1001/jamanetworkopen.2021.34315

**Published:** 2021-11-12

**Authors:** Elena Frank, Zhuo Zhao, Yu Fang, Lisa S. Rotenstein, Srijan Sen, Constance Guille

**Affiliations:** 1 Michigan Neuroscience Institute, University of Michigan, Ann Arbor; 2 Department of Medicine, Brigham and Women’s Hospital, Boston, Massachusetts; 3 Harvard Medical School, Boston, Massachusetts; 4 Department of Psychiatry, University of Michigan Medical School, Ann Arbor; 5 Department of Psychiatry and Behavioral Sciences, Medical University of South Carolina, Charleston

## Abstract

**Importance:**

The COVID-19 pandemic has placed increased strain on health care workers and disrupted childcare and schooling arrangements in unprecedented ways. As substantial gender inequalities existed in medicine before the pandemic, physician mothers may be at particular risk for adverse professional and psychological consequences.

**Objective:**

To assess gender differences in work-family factors and mental health among physician parents during the COVID-19 pandemic.

**Design, Setting, and Participants:**

This prospective cohort study included 276 US physicians enrolled in the Intern Health Study since their first year of residency training. Physicians who had participated in the primary study as interns during the 2007 to 2008 and 2008 to 2009 academic years and opted into a secondary longitudinal follow-up study were invited to complete an online survey in August 2018 and August 2020.

**Exposures:**

Work-family experience included 3 single-item questions and the Work and Family Conflict Scale, and mental health symptoms included the Patient Health Questionnaire–9 (PHQ-9) and Generalized Anxiety Disorder–7 scale.

**Main Outcomes and Measures:**

The primary outcomes were work-to-family and family-to-work conflict and depressive symptoms and anxiety symptoms during August 2020. Depressive symptoms between 2018 (before the COVID-19 pandemic) and 2020 (during the COVID-19 pandemic) were compared by gender.

**Results:**

Among 215 physician parents who completed the August 2020 survey, 114 (53.0%) were female and the weighted mean (SD) age was 40.1 (3.57) years. Among physician parents, women were more likely to be responsible for childcare or schooling (24.6% [95% CI, 19.0%-30.2%] vs 0.8% [95% CI, 0.01%-2.1%]; *P* < .001) and household tasks (31.4% [95% CI, 25.4%-37.4%] vs 7.2% [95% CI, 3.5%-10.9%]; *P* < .001) during the pandemic compared with men. Women were also more likely than men to work primarily from home (40.9% [95% CI, 35.1%-46.8%] vs 22.0% [95% CI, 17.2%-26.8%]; *P* < .001) and reduce their work hours (19.4% [95% CI, 14.7%-24.1%] vs 9.4% [95% CI, 6.0%-12.8%]; *P* = .007). Women experienced greater work-to-family conflict (β = 2.79; 95% CI, 1.00 to 4.59; *P* = .03), family-to-work conflict (β = 3.09; 95% CI, 1.18-4.99; *P* = .02), and depressive (β = 1.76; 95% CI, 0.56-2.95; *P* = .046) and anxiety (β = 2.87; 95% CI, 1.49-4.26; *P* < .001) symptoms compared with men. We observed a difference between women and men in depressive symptoms during the COVID-19 pandemic (mean [SD] PHQ-9 score: 5.05 [6.64] vs 3.52 [5.75]; *P* = .009) that was not present before the pandemic (mean [SD] PHQ-9 score: 3.69 [5.26] vs 3.60 [6.30]; *P* = .86).

**Conclusions and Relevance:**

This study found significant gender disparities in work and family experiences and mental health symptoms among physician parents during the COVID-19 pandemic, which may translate to increased risk for suicide, medical errors, and lower quality of patient care for physician mothers. Institutional and public policy solutions are needed to mitigate the potential adverse consequences for women’s careers and well-being.

## Key Points

**Question:**

Has the COVID-19 pandemic been associated with differences in careers and mental health between physician mothers and fathers?

**Findings:**

In this cohort study of 276 physicians during the COVID-19 pandemic, mothers were more likely than fathers to be responsible for childcare or schooling and household tasks, to work primarily from home, to reduce their work hours, and to experience work-to-family conflict, family-to-work conflict, and depressive and anxiety symptoms. A gender difference in depressive symptoms was observed among physician parents during the COVID-19 pandemic that was not present before the pandemic.

**Meaning:**

This study suggests that pandemic conditions are associated with an increase in gender inequalities within medicine and signals the importance of further attention and resources to mitigate the potential adverse consequences for the careers and well-being of physician mothers.

## Teaser

This cohort study evaluates the association of the COVID-19 pandemic with gender differences in work-family factors and mental health among physician parents.

## Introduction

With more than 100 million cases and 3 million deaths globally,^1^ the COVID-19 pandemic has reshaped nearly every aspect of daily life. For physicians and their families, the increased strain on the US health care system and prolonged childcare and schooling disruptions have created unprecedented professional and personal challenges. Physicians and medical professional organizations have described heightened struggles to balance work and family responsibilities under pandemic conditions and have voiced concern over the potential harmful psychological and career consequences, particularly for female physicians.^2,3^ However, data on the gendered implications of the pandemic among physicians based on validated scientific measures remain scarce.^4^

Even before the COVID-19 pandemic, female physicians faced considerable barriers to achieving gender parity in career advancement^5^ and compensation.^6^ An emerging body of literature suggests that gender disparities in work and family roles and conflict between those roles may be an important factor,^7,8,9,10^ particularly for women in dual-physician families.^11,12^ Pandemic changes in mental and physical demands at work and at home may exacerbate these existing gender inequalities and may therefore pose a greater threat to the career progression of female physicians, especially mothers. At the same time, as frontline health care workers during an infectious disease outbreak, physicians face unique stressors that place them at greater risk for worsening mental health.^13,14,15^ As work-family conflict was previously shown to be associated with depression,^16^ burnout,^11,17^ and emotional exhaustion^18^ for female physicians, mothers might be at particular risk for experiencing adverse psychological effects with increased pandemic work and family stress.

Although early evidence points to the presence of a gender gap in research productivity among academics in general^19,20^ and recent cross-sectional studies suggest high levels of job concern^12,21^ and anxiety among physician mothers,^22^ to our knowledge, the differential effect of the COVID-19 pandemic on psychological well-being has not yet been evaluated longitudinally among physician parents. Given the preexisting gender disparities within medicine and the marked increase in household and caregiving responsibilities under pandemic conditions, investigation of the gendered work-family and mental health experiences of physicians during this period and the ways in which existing drivers of inequity may be exacerbated during such crises is critical to understand potential implications for gender equity within medicine and inform intervention efforts for the current situation as well as mitigation strategies for the future.^23^ The current study sought to address this knowledge gap by assessing work-family factors and mental health symptoms among physician mothers and fathers during the COVID-19 pandemic using survey data collected from a national longitudinal cohort of early-career US physicians.

## Methods

### Study Design and Participants

This cohort study included US physicians enrolled in the Intern Health Study, a prospective cohort study assessing stress and depression during the first year of residency training in the US composed of roughly equal proportions of women and men.^24^ Physicians who had participated in the primary study as interns during the 2007 to 2008 and 2008 to 2009 academic years and opted into a secondary longitudinal follow-up study were invited to complete an online survey in August 2018 and August 2020. All participants provided informed consent via the website and were compensated $25. The University of Michigan institutional review board approved the study. This study followed the Strengthening the Reporting of Observational Studies in Epidemiology (STROBE) reporting guideline

### Study Measures

Participants reported general demographic and work information, including partner’s occupation and employment status. To evaluate work and family experiences during the COVID-19 pandemic, respondents were asked to indicate whether (1) they experienced a loss of childcare or a school closure, (2) they or their partner worked primarily from home, or (3) they or their partner voluntarily reduced their work hours. Participants were also asked who was primarily responsible for (1) childcare or schooling and (2) day-to-day household tasks.

To evaluate the extent that family interfered with work roles and responsibilities (family-to-work conflict) and work interfered with family roles and responsibilities (work-to-family conflict), participants were asked to complete the Work and Family Conflict Scale.^25^ Respondents rated their level of agreement with each item on a 7-point Likert scale from 1 (very strongly disagree) to 7 (very strongly agree). Scores range from 5 to 35 points. The Work and Family Conflict Scale has strong concurrent validity and demonstrates high internal consistency (>.90) and reliability.

To assess depressive symptoms, participants were asked to complete the Patient Health Questionnaire–9 (PHQ-9).^26^ Individuals rated items on a 4-point Likert scale from 0 (not at all) to 3 (nearly every day). Total scores range from 0 to 27. A score of 10 or greater on the PHQ-9 has a sensitivity of 88% and a specificity of 88% for the diagnosis of major depression.

To assess anxiety symptoms, participants were asked to complete the Generalized Anxiety Disorder 7-item scale (GAD-7).^27^ Participants rated items on a 4-point Likert scale from 0 (not at all) to 3 (nearly every day). Total scores range from 0 to 21. The GAD-7 has 89% sensitivity and 82% specificity for detecting clinical generalized anxiety disorder.

### Statistical Analysis

We used a sample weighting strategy to reduce the potential bias introduced from differences between participants and nonparticipants and between participants who completed the annual 2020 survey and those who did not. To reduce potential bias owing to sampling in estimating the changes over time in all factors of interest, we used data from the Association of American Medical Colleges (AAMC) on the overall characteristics of first-year residents in the US in 2007 and 2008 as the reference target and performed a 2-step poststratification ranking on the study data to construct weights such that the distribution of cohort year, sex, specialty, and race and ethnicity in the sample matched the AAMC data distribution. The first step was to generate between-cohort weights (w1b) with the raking variable to be cohort year; the second step was to generate weights within each cohort (w1w) with the raking variables to be sex, specialty, and race and ethnicity. The poststratification rankings were performed with the R package anesrake (R, version 4.1.1 [R Project for Statistical Computing]).^28^ To account for differences between participants who completed the 2020 annual survey and those who did not, we used variables correlated with survey completion to calculate probability of participation using the PSMATCH SAS procedure (SAS, version 9.4 [SAS Institute]) with variables correlated with the completion. The attrition weights (w2) for each person who completed the 2020 annual survey was the inverse of the probability of participation. The final weights were the product of poststratification weights and attrition weights (w1b × w1w × w2).

All analyses were restricted to physicians who had completed their medical training. To compare work and family experiences between physician parents by gender, we conducted a series of weighted χ^2^ tests. To assess the association of different family circumstances with the potential to affect parental burden, we conducted sequential analyses on increasingly restricted samples. First, we assessed the sample of physicians who had at least 1 child younger than 18 years and compared female and male physicians without any children as a control. Then, we restricted the analysis to the subset of physician parents who were working full-time before the COVID-19 pandemic with a full-time physician partner. To account for potential differences in household and childcare responsibilities based on child age, we included the age of the youngest child (<6 years vs ≥6 years) as a covariate in the analysis. We constructed 95% CIs for proportions from binary data using the exact binomial test. To adjust for multiple comparisons, the significance level of 2-sided *α* < .05 was corrected according to the Bonferroni procedure.

To identify factors associated with work-family conflict and anxiety and depressive symptoms, we constructed a series of multivariable weighted regression models with the following participant characteristics: gender, age of the youngest child, number of children, sleep hours during the past week, weekly work hours during the past month, partner employment status, partner occupation, and specialty. Categorical variables were modeled as indicator variables with a reference category.

To assess whether gender differences in depressive symptoms emerged among physician parents during the pandemic, we compared gender differences in PHQ-9 scores between 2018 (before the COVID-19 pandemic) and 2020 (during the COVID-19 pandemic) using the weighted *t* test, limiting the analysis to participants who were parents in both years (n = 180). All analyses were performed using SAS, version 9.4. Two-sided *P* values <.05 were considered statistically significant.

## Results

### Participant Characteristics

The analytic sample included 215 physicians who completed the August 2020 survey. The weighted mean (SD) participant age was 40.1 (3.57), and 114 participants (53.0%) were women. Men were more likely than women to be married, have 3 or more children, work in a surgical specialty (surgical specialties were assigned based on the American College of Surgeons classification^29^), work full time before the COVID-19 pandemic, and have a partner who did not currently work full time (Table 1 and eTable in the Supplement).

**Table 1.** Characteristics of Physician Parents After Sample Weighting

Characteristic	No. (%)		*P* value^a^
	Women (n = 271)	Men (n = 286)	
Age, mean (SD), y	39.9 (3.1)	40.4 (4.0)	.12
Relationship status			
Single	2 (0.8)	0	<.001
In a committed relationship	11 (4.2)	11 (3.8)	
Engaged	0 (0.0)	7 (2.6)	
Married	235 (86.5)	261 (91.2)	
Separated, divorced, or widowed	23 (8.5)	7 (2.4)	
Children, No.			
1	63 (23.4)	45 (15.9)	<.001
2	151 (55.7)	137 (48.1)	
≥3	57 (20.9)	103 (36.1)	
Pre–COVID-19 employment^b^^,^^c^			
Full time	190 (73.1)	248 (91.2)	<.001
Part time	70 (26.9)	24 (8.8)	
Partner’s current employment^b^			
Full time	194 (71.3)	116 (40.4)	<.001
Part time	28 (10.2)	77 (26.9)	
Not employed	50 (18.5)	93 (32.7)	
Partner’s profession			
Physician	95 (35.0)	98 (34.4)	.90
Nonphysician	176 (65.0)	187 (65.6)	
Specialty^d^			
Surgical	40 (14.8)	66 (23.5)	.009
Nonsurgical	230 (85.2)	214 (76.5)	

^a^ *P* value for comparison of women and men.

^b^ Full time was considered 40 hours or more, and part time, less than 40 hours.

^c^ Pre–COVID-19 employment categories (full time, part time) were defined based on mean reported work hours for January 2020 and February 2020.

^d^ Surgical specialties were assigned based on the American College of Surgeons classification.^29^ In this study, physicians in the following specialties were classified as surgical: general surgery, gynecology and obstetrics, neurological surgery, orthopedic surgery, otolaryngology, plastic surgery, urology, and other surgical. Physicians from the following specialties were classified as nonsurgical: internal medicine, pediatrics, psychiatry, neurology, emergency medicine, internal medicine–pediatrics, family medicine, family practice, anesthesiology, dermatology, medical genetics, nuclear medicine, pathology, physical medicine and rehabilitation, preventative medicine, radiation oncology, radiology-diagnostic, sleep medicine, and other nonsurgical.

In 2007 and 2008, 740 of 1271 invited interns (58.2%) enrolled in the primary study. Of these participants, 507 of 740 (68.5%) opted into an additional longitudinal follow-up arm. Between 2009 and 2020, 80 participants (15.8%) were lost to follow up. In total, 276 of 427 contacted physicians (65.1%) completed the August 2020 survey. Compared with all participants in 2007 and 2008, respondents in 2020 were slightly younger (39.8 years vs 40.4 years; *P* = .002) but did not differ significantly from nonrespondents by gender or specialty. Of the participants, 4 (1.4%) had not completed their medical training, and 57 (20.7%) did not currently have a child younger than 18 years and were therefore excluded from the analysis.

### Family and Work Experiences During the COVID-19 Pandemic

**Figure 1.**
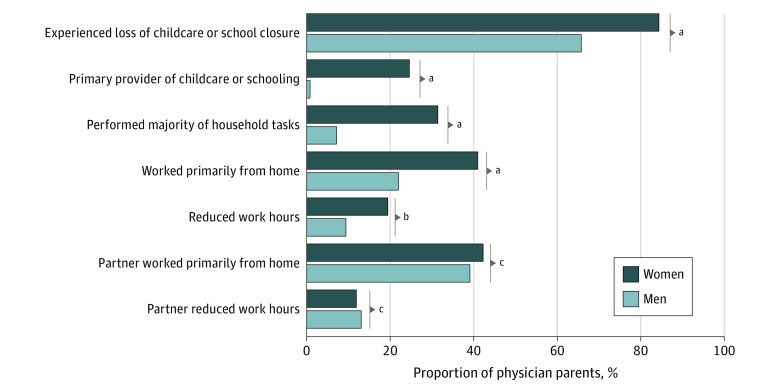
Family and Work Experiences of Physician Parents During the COVID-19 Pandemic by Gender ^a^*P* < .001. ^b^*P* = .007. ^c^*P* > .99.

After accounting for sampling weight and the age of the youngest child, among physician parents, women were significantly more likely to lose childcare during the COVID-19 pandemic compared with men (84.3% [95% CI, 80.0%-88.6%] vs 65.7% [95% CI, 60.2%-71.2%]; *P* < .001) (Figure 1). Among women, 24.6% (95% CI, 19.0%-30.2%] reported that they were primarily responsible for providing childcare or schooling compared with 0.8% (95% CI, 0.01%-2.1%) of men (*P* < .001). Women were also significantly more likely to perform the majority of day-to-day household tasks than were men (31.4% [95% CI, 25.4%-37.4%] vs 7.2% [95% CI, 3.5%-10.9%]; *P* < .001). Among physicians working full-time with full-time physician partners, the gender differences in household responsibilities (44.9% [95% CI, 31.5%-58.4%] vs 4.8% [95% CI, 0.0%-11.2%]; *P* = .004) and childcare and schooling (28.6% [95% CI, 16.3%-40.8%] vs 0%; *P* = .001) were even greater than in the overall samples.

Women were twice as likely to primarily work from home (40.9% [95% CI, 35.1%-46.8%] vs 22.0% [95% CI, 17.2%-26.8%]; *P* < .001) and to voluntarily reduce their work hours (19.4% [95% CI, 14.7%-24.1%] vs 9.4% [95% CI, 6.0%-12.8%]; *P* = .007) during the pandemic compared with men. Women with full-time physician partners were even more likely to primarily work from home (65.0% [95% CI, 52.8%-77.3%] vs 24.6% [95% CI, 13.9%-35.3%]; *P* < .001) and reduce their work hours (25.7% [95% CI, 14.5%-36.9%] vs 2.6% [95% CI, 0.0%-6.6%]; *P* = .007) than were men with full-time physician partners. No significant gender differences in having a work from home partner or partners reducing work hours were observed.

### Work and Family Conflict

In a weighted model accounting for specialty, number of children, age of the youngest child, and partner’s employment status, mothers reported greater work-to-family (β = 2.79; 95% CI, 1.00-4.59; *P* = .03) and family-to-work (β = 3.09; 95% CI, 1.18-4.99; *P* = .02) conflict (Table 2). In addition, fewer sleep hours were associated with greater work-to-family conflict (β = −1.62; 95% CI, −2.57 to −0.67; *P* = .01).

**Table 2.** Multivariable Models of Work-to-Family Conflict and Family-to-Work Conflict Among Physician Parents

Characteristic	Work-to-family conflict		Family-to-work conflict	
	β (95% CI)	*P* value	β (95% CI)	*P* value
Intercept	26.46 (18.41 to 34.51)	<.001	12.75 (4.20 to 21.31)	.04
Gender				
Women	2.79 (1.00 to 4.59)	.03	3.09 (1.18 to 4.99)	.02
Men	1 [Reference]	NA	1 [Reference]	NA
Specialty^a^				
Surgical	1.99 (−0.19 to 4.17)	.88	0.74 (−1.58 to 3.05)	>.99
Nonsurgical	1 [Reference]	NA	1 [Reference]	NA
Children, No.				
1	1 [Reference]	NA	1 [Reference]	NA
2	1.16 (−1.15 to 3.48)	>.99	0.08 (−2.38 to 2.54)	>.99
≥3	1.06 (−1.46 to 3.60)	>.99	0.90 (−1.79 to 3.58)	>.99
Age of youngest child, y				
≤2	0.06 (−2.17 to 2.29)	>.99	1.44 (−0.93 to 3.80)	>.99
3-5	−1.35 (−3.48 to 0.79)	>.99	0.45 (−1.81 to 2.72)	>.99
≥6	1 [Reference]	NA	1 [Reference]	NA
Partner’s employment^b^				
Full time	−0.79 (−3.09 to 1.50)	>.99	−1.26 (−3.70 to 1.17)	>.99
Part time	1.11 (−1.49 to 3.71)	>.99	0.03 (−2.72 to 2.79)	>.99
Not employed	1 [Reference]	NA	1 [Reference]	NA
Partner’s profession				
Physician	0.28 (−1.65 to 2.20)	>.99	1.90 (−0.14 to 3.95)	.81
Nonphysician	1 [Reference]	NA	1 [Reference]	NA
Sleep hours in past week	−1.62 (−2.57 to −0.67)	.01	−0.43 (−1.44 to 0.58)	>.99
Work hours in past month	0.07 (0.01 to 0.12)	.18	0.06 (0.004 to 0.12)	.42

Abbreviation: NA, Not applicable.

^a^ Surgical specialties were assigned based on the American College of Surgeons classification.^29^ In this study, physicians in the following specialties were classified as surgical: general surgery, gynecology and obstetrics, neurological surgery, orthopedic surgery, otolaryngology, plastic surgery, urology, and other surgical. Physicians from the following specialties were classified as nonsurgical: internal medicine, pediatrics, psychiatry, neurology, emergency medicine, internal medicine–pediatrics, family medicine, family practice, anesthesiology, dermatology, medical genetics, nuclear medicine, pathology, physical medicine and rehabilitation, preventative medicine, radiation oncology, radiology-diagnostic, sleep medicine, and other nonsurgical.

^b^ Full time was considered 40 hours or more, and part time, less than 40 hours.

### Mental Health

In a weighted model accounting for specialty, number of children, age of the youngest child, and partner’s employment status, mothers reported greater PHQ-9 depressive symptoms (β = 1.76; 95% CI, 0.56-2.95; *P* = .046) and GAD-7 anxiety symptom scores (β = 2.87; 95% CI, 1.49-4.26; *P* < .001) (Table 3). Fewer sleep hours were also associated with an increase in PHQ-9 score (β = −1.19; 95% CI, −1.82 to −0.56; *P* = .003) and GAD-7 score (β = −1.13; 95% CI, −1.86 to −0.40; *P* = .03).

**Table 3.** Multivariable Models of Depressive and Anxiety Symptoms Among Physician Parents

Characteristic	PHQ-9 depressive symptoms		GAD-7 anxiety symptoms	
	β (95% CI)	*P* value	β (95% CI)	*P* value
Intercept	10.91 (5.74 to 16.08)	<.001	9.80 (3.81 to 15.77)	.02
Gender				
Women	1.76 (0.56 to 2.95)	.05	2.87 (1.49 to 4.26)	<.001
Men	1 [Reference]	NA	1 [Reference]	NA
Specialty^a^				
Surgical	0.72 (−0.73 to 2.17)	>.99	0.58 (−1.10 to 2.26)	>.99
Nonsurgical	1 [Reference]	NA	1 [Reference]	NA
Children, No.				
1	1 [Reference]	NA	1 [Reference]	NA
2	0.69 (−1.00 to 2.37)	>.99	1.36 (−0.59 to 3.31)	>.99
≥3	0.30 (−0.98 to 1.58)	>.99	0.51 (−0.97 to 1.99)	>.99
Age of youngest child, y				
≤2	0.75 (0.57 to 2.08)	>.99	1.04 (−0.49 to 2.57)	>.99
3-5	0.91 (−0.58 to 2.39)	>.99	0.75 (−0.97 to 2.47)	>.99
≥6	1 [Reference]	NA	1 [Reference]	NA
Partner’s employment^b^				
Full time	−0.89 (−2.42 to 0.64)	>.99	−0.36 (−2.13 to 1.41)	>.99
Part time	1.10 (−0.63 to 2.83)	>.99	1.69 (−0.32 to 3.69)	>.99
Not employed	1 [Reference]	NA	1 [Reference]	NA
Partner’s profession				
Physician	0.18 (−1.11 to 1.46)	>.99	0.26 (−1.23 to 1.74)	>.99
Nonphysician	1 [Reference]	NA	1 [Reference]	NA
Sleep hours in past week	−1.19 (−1.82 to −0.56)	.003	−1.13 (−1.86 to −0.40)	.03
Work hours in past month	0.002 (−0.03 to 0.04)	>.99	−0.0004 (−0.04 to 0.04)	>.99

Abbreviations: GAD-7, Generalized Anxiety Disorder 7-item scale; NA, not applicable; PHQ-9, Patient Health Questionnaire–9.

^a^ Surgical specialties were assigned based on the American College of Surgeons classification.^29^ In this study, physicians in the following specialties were classified as surgical: general surgery, gynecology and obstetrics, neurological surgery, orthopedic surgery, otolaryngology, plastic surgery, urology, and other surgical. Physicians from the following specialties were classified as nonsurgical: internal medicine, pediatrics, psychiatry, neurology, emergency medicine, internal medicine–pediatrics, family medicine, family practice, anesthesiology, dermatology, medical genetics, nuclear medicine, pathology, physical medicine and rehabilitation, preventative medicine, radiation oncology, radiology-diagnostic, sleep medicine, and other nonsurgical.

^b^ Full time was considered 40 hours or more, and part time, less than 40 hours.

**Figure 2.**
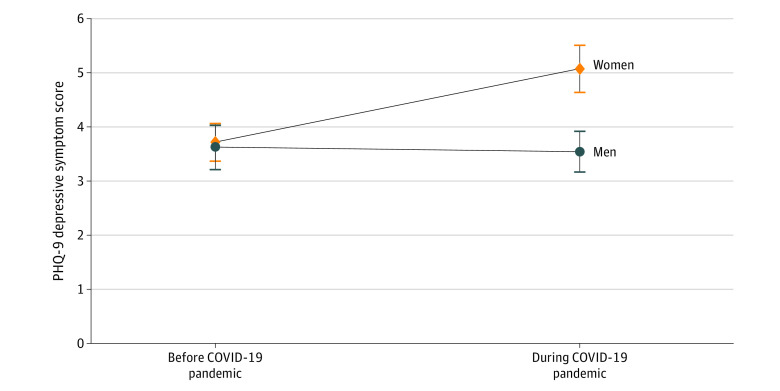
Patient Health Questionnaire–9 (PHQ-9) Depressive Symptoms Among Physician Parents Before and During the COVID-19 Pandemic by Gender Mean PHQ-9 depressive symptom scores among the subset of the sample assessed in 2018 and 2020 with children in both years (n = 180). In August 2018 (before the COVID-19 pandemic), no statistically significant gender difference in PHQ-9 scores was found (*P* = .86). In contrast, in August 2020 (during the COVID-19 pandemic) PHQ-9 scores were significantly higher for women than for men (*P* = .009).

To assess whether the parental gender difference in PHQ-9 depressive symptoms predated the pandemic, we focused on the subset of the sample who were also assessed in 2018 and were parents at that time (n = 180). Consistent with the full weighted sample, mean (SD) PHQ-9 depressive symptom scores in 2020 were significantly higher for women compared with men (5.05 [6.64] vs 3.52 [5.75]; *P* = .009). In contrast, there was no statistically significant gender difference in mean (SD) PHQ-9 scores found in this cohort in 2018 (women vs men: 3.69 [5.26] vs 3.60 [6.30]; *P* = .86) (Figure 2). Among individuals who were not parents, women and men did not differ significantly in work and family experiences or conflict, PHQ-9 depression scores, or GAD-7 anxiety scores.

## Discussion

Findings from this national cohort study of early-career US physicians suggest that the COVID-19 pandemic is associated with an increase in existing work-family balance struggles for physician mothers, adversely affecting their professional lives and psychological well-being. Of note, we observed a significant gender difference in depression that was not present before the COVID-19 pandemic.

Parenthood has previously been associated with reduced work hours, income, and retention for women in medicine, with female physicians more likely to be part of a dual-career family and to experience greater work-family balance challenges.^10,30,31,32,33^ Consistent with other professions, female physicians tend to perform a greater share of domestic and caregiving duties than male physicians and are 3 times as likely to sacrifice work time to care for their children.^7,34^ During the COVID-19 pandemic, we identified an even greater disparity, with physician mothers 30 times more likely to manage childcare and schooling than physician fathers. Among dual-physician couples, none of the men reported taking a primary role in managing the increased pandemic caregiving demands.

With their greater overall pandemic workload, we found that women experienced significantly higher levels of conflict between their roles as physicians and parents. To relieve the intensifying pressures of their professional life and “second shift” at home,^35^ in line with recent trends in other fields,^36^ we found that physician mothers disproportionately downshifted at work by reducing their work hours or working primarily from home. Consistent with prior work on dual-physician marriages,^11,12^ we found that women with physician partners were even more likely to make these adjustments during the pandemic.

Work-family conflict has been associated with emotional exhaustion,^18^ burnout,^11,17^ and depression^16^ among female physicians. Under increased pandemic strain, we identified a similar pattern in which physician mothers’ risk for depression increased significantly compared with physician fathers’. Of note, no gender difference in depression risk was present in this group before the COVID-19 pandemic. Although high rates of anxiety have been identified among physician mothers during the pandemic,^22^ this study is the first, to our knowledge, to demonstrate the differential and longitudinal association of the pandemic with the mental health of physician mothers. As physician depression has been associated with increased risk for suicide, medical errors, and lower quality of patient care,^37,38,39^ this has important implications for the well-being of physician mothers as well as their patients.

Given existing gender inequities in medicine, the career stakes of the COVID-19 pandemic for physician mothers are high. Early evidence from the pandemic suggests that women experience more negative professional consequences than men under work-from-home conditions^40^ and lower productivity.^19^ Even short-term adjustments can have serious long-term repercussions as they may lead to lower earnings and negatively impact opportunities for promotion,^41^ further exacerbating gender inequalities in compensation^6^ and advancement.^4^ Although the greater availability of telehealth services has increased work flexibility,^42^ women who are rarely onsite may miss out on critical career opportunities and experience the added strain of simultaneously managing work and childcare at home.

With the increased burden placed on physician mothers during the pandemic, our findings suggest that the existing gender gap in early-career physician retention, which are primarily associated with family factors, will continue to grow.^10^ As some recent studies have found that female physicians spend more time with patients^43^ and have better patient outcomes,^44^ this loss could be devastating to the US health care system, particularly in the context of a global pandemic and an impending physician shortage. For those physician mothers forced out of the workforce, creating a viable path for re-entry into medicine after the pandemic will be critical.

Further institutional and public policy solutions are needed to mitigate the potential adverse effects of the COVID-19 pandemic for women’s careers and well-being and to ensure that recent gender equity gains in medicine are not lost. Given the current gender gaps in pay and promotion, institutions should actively work to recruit, retain, and advance women and be vigilant that cost-cutting measures and career advancement metrics do not disproportionately penalize them.^45,46^ Increased support for family care needs, including childcare and paid family leave, as well as wellness programs tailored for physician mothers are vital to protect against adverse effects on productivity and well-being during the pandemic and beyond.^47^ Initiatives to increase work schedule and location flexibility are also critical.^45^ In addition, as the stark gender differences in pandemic experiences identified among dual-physician couples have shown, a shift in the culture around work and family balance in medicine is needed. Efforts to normalize the use of parental leave and sick days among male physicians, for example, could help challenge existing gender biases in work and family expectations. An initiative similar to #TimesUp in health care that aims to increase awareness and address inequities related to work-family challenges may be critical to improving workplace conditions and mental health for women.^48^

### Strengths and Limitations

This study has strengths. We used detailed measures and scientifically validated tools to assess gender differences in work-family conflict and mental health during the COVID-19 pandemic. Furthermore, our longitudinal study design facilitated the comparison of depressive symptoms among the same participants before and during the COVID-19 pandemic, which allowed us to assess whether a gender gap in depressive symptoms emerged during the pandemic that was not identified in prior work, to our knowledge. We also minimized the risk of response bias present in many COVID-19 studies by including the COVID-19 experience questions as part of a routine follow-up survey in an existing physician cohort.

This study also has limitations. First, the sample consisted of early-career physicians in the US; thus, the results may not be generalizable to all physicians or to physicians in other countries. Second, although the sample weighting can help to mitigate bias, definitive causal conclusions cannot be drawn from observational studies. It is possible other factors related to participant attrition may have influenced the study results, although we found no meaningful differences in age, gender, or specialty between respondents and those lost to follow-up. Third, because of the limited sample size, the precise magnitude estimates of the group differences identified in this study were not possible. Fourth, all data were obtained via self-report rather than a diagnostic interview, and longitudinal data were not available for family responsibilities, work and family conflict, and anxiety symptoms. Future investigation of the role of other factors, such as sexual orientation, race, ethnicity, immigration status, relationship status, geographical location, and income as well as other personal (eg, elder care responsibilities, social support) and work factors (eg, hours worked at home, partner specialty, and work environment) would be beneficial.

## Conclusions

This cohort study is, to our knowledge, the first to systematically examine gender differences in work-family conflict and well-being in the same cohort of physician parents before and during the COVID-19 pandemic. Findings from this national study suggest that gender disparities within medicine may have increased in association with pandemic work and home conditions, with disproportionate consequences for the mental health and careers of physician mothers. These findings underscore the importance of taking immediate action to ensure women have access to the resources and supports necessary to navigate this unprecedented and uncertain time and the work-family challenges that may ensue moving forward.

## Notes

Supplement. **eTable.** Characteristics of Physician Parents Before Sample Weighting

## References

[1] World Health Organization. Weekly epidemiological update—6 July 2021. 2021. Accessed July 7, 2021. https://www.who.int/publications/m/item/weekly-epidemiological-update-on-covid-19—6-july-2021

[2] Brubaker L. Women physicians and the COVID-19 pandemic. JAMA. 2020;324(9):835-836. doi:10.1001/jama.2020.14797 32735329

[3] Spector ND, Overholser B. COVID-19 and the slide backward for women in academic medicine. JAMA Netw Open. 2020;3(9):e2021061-e2021061. doi:10.1001/jamanetworkopen.2020.21061 32940676

[4] Jagsi R, Fuentes-Afflick E, Higginbotham E. Promoting equity for women in medicine—seizing a disruptive opportunity. N Engl J Med. 2021;384(24):2265-2267. doi:10.1056/NEJMp2104228 34134182

[5] Richter KP, Clark L, Wick JA, . Women physicians and promotion in academic medicine. N Engl J Med. 2020;383(22):2148-2157. doi:10.1056/NEJMsa1916935 33252871

[6] Ly DP, Seabury SA, Jena AB. Differences in incomes of physicians in the United States by race and sex: observational study. BMJ. 2016;353:i2923. doi:10.1136/bmj.i2923 27268490 PMC4897176

[7] Jolly S, Griffith KA, DeCastro R, Stewart A, Ubel P, Jagsi R. Gender differences in time spent on parenting and domestic responsibilities by high-achieving young physician-researchers. Ann Intern Med. 2014;160(5):344-353. doi:10.7326/M13-0974 24737273 PMC4131769

[8] Ly DP, Jena AB. Sex differences in time spent on household activities and care of children among US physicians, 2003-2016. Mayo Clin Proc. 2018;93(10):1484-1487. doi:10.1016/j.mayocp.2018.02.018 29673711 PMC8099448

[9] Lyu HG, Davids JS, Scully RE, Melnitchouk N. Association of domestic responsibilities with career satisfaction for physician mothers in procedural vs non-procedural fields. JAMA Surg. 2019;154(8):689-695. doi:10.1001/jamasurg.2019.0529 30969336 PMC6583845

[10] Frank E, Zhao Z, Sen S, Guille C. Gender disparities in work and parental status among early career physicians. JAMA Netw Open. 2019;2(8):e198340. doi:10.1001/jamanetworkopen.2019.8340 31373646 PMC6681644

[11] Dyrbye LN, Shanafelt TD, Balch CM, Satele D, Sloan J, Freischlag J. Relationship between work-home conflicts and burnout among American surgeons: a comparison by sex. Arch Surg. 2011;146(2):211-217. doi:10.1001/archsurg.2010.310 21339435

[12] Soares A, Thakker P, Deych E, Jain S, Bhayani RK. The impact of COVID-19 on dual-physician couples: a disproportionate burden on women physicians. J Womens Health (Larchmt). 2021;30(5):665-671. doi:10.1089/jwh.2020.8903 33751922 PMC8182652

[13] De Brier N, Stroobants S, Vandekerckhove P, De Buck E. Factors affecting mental health of health care workers during coronavirus disease outbreaks (SARS, MERS & COVID-19): a rapid systematic review. PLoS One. 2020;15(12):e0244052. doi:10.1371/journal.pone.0244052 33320910 PMC7737991

[14] Lai J, Ma S, Wang Y, . Factors associated with mental health outcomes among health care workers exposed to coronavirus disease 2019. JAMA Netw Open. 2020;3(3):e203976-e203976. doi:10.1001/jamanetworkopen.2020.3976 32202646 PMC7090843

[15] Li W, Frank E, Zhao Z, . Mental health of young physicians in China during the novel coronavirus disease 2019 outbreak. JAMA Netw Open. 2020;3(6):e2010705-e2010705. doi:10.1001/jamanetworkopen.2020.10705 32478846 PMC7265093

[16] Guille C, Frank E, Zhao Z, . Work-family conflict and the sex difference in depression among training physicians. JAMA Intern Med. 2017;177(12):1766-1772. doi:10.1001/jamainternmed.2017.5138 29084311 PMC5820732

[17] Langballe EM, Innstrand ST, Aasland OG, Falkum E. The predictive value of individual factors, work-related factors, and work-home interaction on burnout in female and male physicians: a longitudinal study. Stress Health. 2011;27:73-87. doi:10.1002/smi.1321

[18] Ahmad A. Work-family conflict among junior physicians: its mediating role in the relationship between role overload and emotional exhaustion. J Soc Sci. 2010;6(2):265-271. doi:10.3844/jssp.2010.265.271

[19] Vincent-Lamarre P, Sugimoto CR, Larivière V. The Decline of Women’s Research Production During the Coronavirus Pandemic. Nature Index; 2020:19.

[20] Krukowski RA, Jagsi R, Cardel MI. Academic productivity differences by gender and child age in science, technology, engineering, mathematics, and medicine faculty during the COVID-19 pandemic. J Womens Health (Larchmt). 2021;30(3):341-347. doi:10.1089/jwh.2020.8710 33216682 PMC7957370

[21] Matulevicius SA, Kho KA, Reisch J, Yin H. Academic medicine faculty perceptions of work-life balance before and since the COVID-19 pandemic. JAMA Netw Open. 2021;4(6):e2113539. doi:10.1001/jamanetworkopen.2021.13539 34129021 PMC8207238

[22] Linos E, Halley MC, Sarkar U, . Anxiety levels among physician-mothers during the COVID pandemic. Am J Psychiatry. 2021;178(2):203-204. doi:10.1176/appi.ajp.2020.20071014 33517747

[23] National Academies of Sciences, Engineering, and Medicine. Impact of COVID-19 on the Careers of Women in Academic Sciences, Engineering, and Medicine. National Academies Press; 2021. 33705087

[24] Sen S, Kranzler HR, Didwania AK, . Effects of the 2011 duty hour reforms on interns and their patients: a prospective longitudinal cohort study. JAMA Intern Med. 2013;173(8):657-662. doi:10.1001/jamainternmed.2013.351 23529201 PMC4016974

[25] Haslam D, Filus A, Morawska A, Sanders MR, Fletcher R. The Work-Family Conflict Scale (WAFCS): development and initial validation of a self-report measure of work-family conflict for use with parents. Child Psychiatry Hum Dev. 2015;46(3):346-357. doi:10.1007/s10578-014-0476-0 24919779

[26] Kroenke K, Spitzer RL, Williams JB. The PHQ-9: validity of a brief depression severity measure. J Gen Intern Med. 2001;16(9):606-613. doi:10.1046/j.1525-1497.2001.016009606.x 11556941 PMC1495268

[27] Spitzer RL, Kroenke K, Williams JB. Validation and utility of a self-report version of PRIME-MD: the PHQ primary care study. JAMA. 1999;282(18):1737-1744. doi:10.1001/jama.282.18.1737 10568646

[28] DeBell M. Best practices for creating survey weights. In: Vannette D, Krosnick J, eds. The Palgrave Handbook of Survey Research. Palgrave Macmillan; 2018:159-162. doi:10.1007/978-3-319-54395-6_21

[29] American College of Surgeons. Surgical specialties. Accessed June 10, 2021. http://www.facs.org/member-services/join/specialties

[30] Jacobson CC, Nguyen JC, Kimball AB. Gender and parenting significantly affect work hours of recent dermatology program graduates. Arch Dermatol. 2004;140(2):191-196. doi:10.1001/archderm.140.2.191 14967791

[31] Grant L, Simpson LA, Rong XL, Peters-Golden H. Gender, parenthood, and work hours of physicians. J Marriage Fam. 1990;52:39-49. doi:10.2307/352836

[32] Hinze SW. Inside medical marriages: the effect of gender on income. Work Occup. 2000;27(4):464-499. doi:10.1177/0730888400027004003

[33] Sasser AC. Gender differences in physician pay tradeoffs between career and family. J Hum Resour. 2005;40(2):477-504. doi:10.3368/jhr.XL.2.477

[34] Bianchi SM, Sayer LC, Milkie MA, Robinson JP. Housework: who did, does or will do it, and how much does it matter? Soc Forces. 2012;91(1):55-63. doi:10.1093/sf/sos120 25429165 PMC4242525

[35] Hochschild A, Machung A. The Second Shift: Working Families and the Revolution At Home. Penguin; 2012.

[36] McKinsey and Co. Women in the workplace. 2020. Accessed January 14, 2021. https://www.mckinsey.com/featured-insights/diversity-and-inclusion/women-in-the-workplace

[37] Tyssen R, Vaglum P, Grønvold NT, Ekeberg O. Suicidal ideation among medical students and young physicians: a nationwide and prospective study of prevalence and predictors. J Affect Disord. 2001;64(1):69-79. doi:10.1016/S0165-0327(00)00205-6 11292521

[38] Schernhammer ES, Colditz GA. Suicide rates among physicians: a quantitative and gender assessment (meta-analysis). Am J Psychiatry. 2004;161(12):2295-2302. doi:10.1176/appi.ajp.161.12.2295 15569903

[39] Fahrenkopf AM, Sectish TC, Barger LK, . Rates of medication errors among depressed and burnt out residents: prospective cohort study. BMJ. 2008;336(7642):488-491. doi:10.1136/bmj.39469.763218.BE 18258931 PMC2258399

[40] Center for American Progress. Valuing women’s caregiving during and after the coronavirus crisis. Accessed January 14, 2021. https://www.americanprogress.org/issues/women/reports/2020/06/03/485855/valuing-womens-caregiving-coronavirus-crisis/

[41] Freund KM, Raj A, Kaplan SE, . Inequities in academic compensation by gender: a follow-up to the National Faculty Survey Cohort Study. Acad Med. 2016;91(8):1068-1073. doi:10.1097/ACM.0000000000001250 27276007 PMC4965349

[42] Wamsley CE, Kramer A, Kenkel JM, Amirlak B. Trends and challenges of telehealth in an academic institution: the unforeseen benefits of the COVID-19 global pandemic. Aesthet Surg J. 2021;41(1):109-118. doi:10.1093/asj/sjaa212 32697289 PMC7543904

[43] Ganguli I, Sheridan B, Gray J, Chernew M, Rosenthal MB, Neprash H. Physician work hours and the gender pay gap—evidence from primary care. N Engl J Med. 2020;383(14):1349-1357. doi:10.1056/NEJMsa2013804 32997909 PMC10854207

[44] Tsugawa Y, Jena AB, Figueroa JF, Orav EJ, Blumenthal DM, Jha AK. Comparison of hospital mortality and readmission rates for Medicare patients treated by male vs female physicians. JAMA Intern Med. 2017;177(2):206-213. doi:10.1001/jamainternmed.2016.7875 27992617 PMC5558155

[45] Salles A, Jagsi R. Institutional imperatives for the advancement of women in medicine and science through the COVID-19 pandemic. Lancet. 2021;398(10304):937-939. doi:10.1016/S0140-6736(21)01912-7 34450081 PMC8455349

[46] Narayana S, Roy B, Merriam S, ; on behalf of the Society of General Internal Medicine’s Women and Medicine Commission. Minding the gap: organizational strategies to promote gender equity in academic medicine during the COVID-19 pandemic. J Gen Intern Med. 2020;35(12):3681-3684. doi:10.1007/s11606-020-06269-0 33021718 PMC7537583

[47] Das D, Lall MD, Walker L, Dobiesz V, Lema P, Agrawal P. The multifaceted impact of COVID-19 on the female academic emergency physician: a national conversation. AEM Educ Train. 2020;5(1):91-98. doi:10.1002/aet2.10539 33553984 PMC7849338

[48] Choo EK, Byington CL, Johnson NL, Jagsi R. From #MeToo to #TimesUp in health care: can a culture of accountability end inequity and harassment? Lancet. 2019;393(10171):499-502. doi:10.1016/S0140-6736(19)30251-X 30739670

