# Supplementary material for: Experiences of Work-Family Conflict and Mental Health Symptoms by Gender Among Physician Parents During the COVID-19 Pandemic
Source: JAMA Netw Open. 2021 Nov 12;4(11):e2134315. doi: 10.1001/jamanetworkopen.2021.34315 (PMC8590168; doi:10.1001/jamanetworkopen.2021.34315)
Supplement: Supplement. — eTable. Characteristics of Physician Parents Before Sample Weighting [file jamanetwopen-e2134315-s001.pdf]

# Supplemental Online Content

Frank E, Zhao Z, Fang Y, Rotenstein LS, Sen S, Guille C. Experiences of work-family conflict and mental health symptoms by gender among physician parents during the COVID-19 pandemic. *JAMA Netw Open.* 2021;4(11):e2134315. doi:10.1001/jamanetworkopen.2021.34315

**eTable.** Characteristics of Physician Parents Before Sample Weighting

This supplemental material has been provided by the authors to give readers additional information about their work.

© 2021 Frank E et al. *JAMA Network Open.*

eTable. Characteristics of Physician Parents Before Sample Weighting

| | Women (n=114) | Men (n=101) | P Value[a] |
|---|---|---|---|
| Mean age (SD), y | 39.6 (2.0) | 39.8 (2.2) | 0.39 |
| Relationship status, n (%) | | | .089 |
| Single | 1 (0.9) | 0 (0.0) | |
| In a committed relationship | 5 (4.4) | 4 (4.0) | |
| Engaged | 0 (0.0) | 3 (3.0) | |
| Married | 99 (86.8) | 92 (91.0) | |
| Separated / Divorced / Widowed | 9 (7.9) | 2 (2.0) | |
| Number of children, n (%) | | | .072 |
| 1 | 27 (23.7) | 17 (16.8) | |
| 2 | 62 (54.4) | 48 (47.5) | |
| ≥3 | 25 (22.0) | 36 (35.6) | |
| Pre-COVID-19 employment, n (%) | | | <.001 |
| Full-time (≥ 40 h) | 77 (67.5) | 88 (87.1) | |
| Part-time (< 40 h) | 31 (27.2) | 7 (6.9) | |
| Missing | 6 (5.3) | 6 (6.0) | |
| Partner's current employment[b], n (%) | | | <.001 |
| Full-time | 83 (72.8) | 40 (39.6) | |
| Part-time | 10 (8.8) | 25 (24.8) | |
| Not employed | 21 (18.4) | 36 (35.6) | |
| Partner's profession, n (%) | | | .95 |
| Physician | 39 (34.2) | 35 (34.7) | |
| Non-physician | 75 (65.8) | 66 (65.4) | |
| Specialty[50,c], n (%) | | | .084 |
| Surgical | 14 (12.3) | 21 (20.8) | |
| Non-surgical | 99 (86.8) | 78 (77.2) | |
| Missing | 1 (0.8) | 2 (2.0) | |

[a]P value for comparison of women and men.

© 2021 Frank E et al. *JAMA Network Open.*

[b]Pre-COVID-19 employment categories (full-time, part-time) were defined based on mean reported work hours for January 2020 and February 2020.

[50, c]Surgical specialties were assigned based on the American College of Surgeons classification. Specifically, for this study, physicians in the following specialties were classified as “surgical”: General Surgery, Gynecology and Obstetrics, Neurological Surgery, Orthopaedic Surgery, Otolaryngology, Plastic Surgery, Urology, and Other surgical. Physicians from the following specialties were classified as “non-surgical”: Internal Medicine, Pediatrics, Psychiatry, Neurology, Emergency Medicine, Internal Medicine-Pediatrics, Family Medicine, Family Practice, Anesthesiology, Dermatology, Medical Genetics, Nuclear Medicine, Pathology, Physical Medicine & Rehabilitation, Preventative Medicine, Radiation Oncology, Radiology-Diagnostic, Sleep Medicine, and Other Non-surgical.

© 2021 Frank E et al. *JAMA Network Open.*

ESURVEY

Current relationship status:

- ❍ Not in a committed relationship
- ❍ In a committed relationship
- ❍ Engaged
- ❍ Married
- ❍ Separated / Divorced / Widowed

What is your partner's current occupation?

- ❍ Student
- ❍ Physician
- ❍ Other medical professional (e.g. dentist, nurse, pharmacist)
- ❍ Non-medical professional (e.g. business person, engineer)
- ❍ Other occupation_____
- ❍ Not a student or working for pay outside the home

What is your partner's current work schedule?

- ❍ Full-time
- ❍ Part-time

How many children do you have?

- ❍ 0
- ❍ 1
- ❍ 2
- ❍ 3 or more

© 2021 Frank E et al. *JAMA Network Open.*

What is your current position?

- ❍ Resident
- ❍ Fellow
- ❍ Out of training
- ❍ Not currently practicing medicine

What is your current employment status?

- ❍ Full-time
- ❍ Part-time
- ❍ Not employed

© 2021 Frank E et al. *JAMA Network Open.*

What is your specialty?

- ❍ Anesthesiology
- ❍ Dermatology
- ❍ Emergency Medicine
- ❍ Family Medicine
- ❍ Family Practice
- ❍ Internal Medicine
- ❍ Internal Medicine-Pediatrics
- ❍ Medical Genetics
- ❍ Neurological Surgery
- ❍ Neurology
- ❍ Nuclear Medicine
- ❍ Obstetrics & Gynecology
- ❍ Ophthalmology
- ❍ Orthopaedic Surgery
- ❍ Otolaryngology
- ❍ Pathology-Anatomic & Clinical
- ❍ Pediatrics
- ❍ Physical Medicine & Rehabilitation
- ❍ Plastic Surgery
- ❍ Preventive Medicine
- ❍ Psychiatry
- ❍ Radiation Oncology
- ❍ Radiology-Diagnostic
- ❍ Sleep Medicine
- ❍ Surgery-General
- ❍ Urology
- ❍ Other

© 2021 Frank E et al. *JAMA Network Open.*

Excluding vacation time, on average how many hours per week have you worked over the PAST YEAR?

Please indicate your average weekly work hours for each month so far this year:

January 2020:

February 2020:

March 2020

April 2020:

May 2020:

June 2020:

July 2020:

On average, how many hours have you slept per night over the PAST WEEK?

© 2021 Frank E et al. *JAMA Network Open.*

MOOD SYMPTOMS For each statement, please mark the response which best represents how often you have been bothered by any of the following problems over the PAST 2 WEEKS

| | Not at all (0) | Less than half the days (1) | More than half the days (2) | Nearly everyday (3) |
|---|---|---|---|---|
| Little interest or pleasure in doing things (1) | ❍ | ❍ | ❍ | ❍ |
| Feeling down, depressed or hopeless (2) | ❍ | ❍ | ❍ | ❍ |
| Trouble falling asleep, staying asleep or sleeping too much (3) | ❍ | ❍ | ❍ | ❍ |
| Feeling tired or having little energy (4) | ❍ | ❍ | ❍ | ❍ |
| Poor appetite or overeating (5) | ❍ | ❍ | ❍ | ❍ |
| Feeling badly about yourself, or that you are a failure, or that you have let yourself or your family down (6) | ❍ | ❍ | ❍ | ❍ |
| Trouble concentrating on things such as reading the newspaper or watching TV (7) | ❍ | ❍ | ❍ | ❍ |
| Moving or speaking so slow that others could have noticed or the opposite, being so fidgety or restless that you have been moving around a lot more than usual (8) | ❍ | ❍ | ❍ | ❍ |
| Thoughts that you would be better off dead or hurting yourself in some way (9) | ❍ | ❍ | ❍ | ❍ |

© 2021 Frank E et al. *JAMA Network Open.*

If you have experienced any of the depressive symptoms described, how difficult have they made it for you to do your work, take care of things at home, or get along with other people?

- ❍ Have not experienced any depressive symptoms
- ❍ Not difficult at all
- ❍ Somewhat difficult
- ❍ Very difficult
- ❍ Extremely difficult

© 2021 Frank E et al. *JAMA Network Open*.

Over the LAST TWO WEEKS, how often have you been bothered by the following problems?

| | Not at all (0) | Less than half the days (1) | More than half the days (2) | Nearly everyday (3) |
|---|---|---|---|---|
| Feeling anxious, nervous, or on edge (1) | ❍ | ❍ | ❍ | ❍ |
| Not being able to stop or control worrying (2) | ❍ | ❍ | ❍ | ❍ |
| Worrying too much about different things (3) | ❍ | ❍ | ❍ | ❍ |
| Trouble relaxing (4) | ❍ | ❍ | ❍ | ❍ |
| Being so restless that it's hard to sit still (5) | ❍ | ❍ | ❍ | ❍ |
| Becoming easily annoyed or irritable (6) | ❍ | ❍ | ❍ | ❍ |
| Feeling afraid as if something awful might happen (7) | ❍ | ❍ | ❍ | ❍ |

© 2021 Frank E et al. *JAMA Network Open.*

WORK AND FAMILY LIFE Please rate how much you agree with the following statements.

| | Very strongly disagree (1) | Strongly disagree (2) | Disagree (3) | Neither agree nor disagree (4) | Agree (5) | Strongly agree (6) | Very strongly agree (7) |
|---|---|---|---|---|---|---|---|
| My work prevents me spending sufficient quality time with my family (1) | ❍ | ❍ | ❍ | ❍ | ❍ | ❍ | ❍ |
| There is no time left at the end of the day to do the things I'd like at home (e.g., chores and leisure activities) (2) | ❍ | ❍ | ❍ | ❍ | ❍ | ❍ | ❍ |
| My family misses out because of my work commitments (3) | ❍ | ❍ | ❍ | ❍ | ❍ | ❍ | ❍ |
| My work has a negative impact on my family life (4) | ❍ | ❍ | ❍ | ❍ | ❍ | ❍ | ❍ |
| Working often makes me irritable or short tempered at home (5) | ❍ | ❍ | ❍ | ❍ | ❍ | ❍ | ❍ |
| My work performance suffers because of my personal and family commitments (6) | ❍ | ❍ | ❍ | ❍ | ❍ | ❍ | ❍ |
| Family related concerns or responsibilities often distract me at work (7) | ❍ | ❍ | ❍ | ❍ | ❍ | ❍ | ❍ |
| If I did not have a family I'd be a better employee (8) | ❍ | ❍ | ❍ | ❍ | ❍ | ❍ | ❍ |
| My family has a negative impact on my day to day work duties (9) | ❍ | ❍ | ❍ | ❍ | ❍ | ❍ | ❍ |
| It is difficult to concentrate at work because I am so exhausted by family responsibilities (10) | ❍ | ❍ | ❍ | ❍ | ❍ | ❍ | ❍ |

Please indicate if you have experienced any of the following during the COVID-19 pandemic:

- ❑ You provided direct care to a patient with known or suspected COVID-19
- ❑ You contracted COVID-19
- ❑ A member of your household contracted COVID-19

© 2021 Frank E et al. *JAMA Network Open.*

- ❑ Your household living arrangements changed to protect others in your household from COVID-19
- ❑ Loss or involuntary reduction of your employment income
- ❑ Loss or involuntary reduction of your partner's employment income
- ❑ Loss of childcare or school closure
- ❑ You worked primarily from home
- ❑ You voluntarily reduced your work hours
- ❑ Your partner worked primarily from home
- ❑ Your partner voluntarily reduced their work hours

[*BRANCH: If "yes" to "loss of child care or school closure", both questions below*]

Who is/was primarily responsible for providing childcare or schooling during this period?

- You
- Your spouse or partner
- A sibling
- Other family (e.g. grandparents, aunts/uncles)
- A paid in-home child care provider (e.g. nanny)
- A child care facility located outside your home
- Other________

Who in your family is/was responsible for performing day-to-day (non-childcare) household tasks during this period (such as cleaning, cooking, laundry, etc.)?

- I performed most or all of the household tasks
- I performed slightly more household tasks than my significant other
- My significant other and I shared the responsibility for performing household tasks evenly
- My significant other performed slightly more household tasks than I do
- My significant other performed most or all of the household tasks
- Other family performed most household tasks
- We employed outside help to aid with most household tasks

© 2021 Frank E et al. *JAMA Network Open.*
